# Spontaneous cognitive processes and the behavioral validation of time-varying brain connectivity

**DOI:** 10.1162/netn_a_00037

**Published:** 2018-10-01

**Authors:** Aaron Kucyi, Arielle Tambini, Sepideh Sadaghiani, Shella Keilholz, Jessica R. Cohen

**Affiliations:** Department of Neurology and Neurological Sciences, Stanford University, Stanford, CA, USA; Department of Psychology, and Helen Wills Neuroscience Institute, University of California, Berkeley, CA, USA; Department of Psychology, and Beckman Institute for Advanced Science and Technology, University of Illinois at Urbana-Champaign, IL, USA; Department of Biomedical Engineering, Emory University/Georgia Institute of Technology, Atlanta, GA, USA; Department of Psychology and Neuroscience, University of North Carolina at Chapel Hill, NC, USA

**Keywords:** Dynamic functional connectivity, Default mode network, Resting state, Spontaneous cognition, Mind wandering, Arousal

## Abstract

In cognitive neuroscience, focus is commonly placed on associating brain function with changes in objectively measured external stimuli or with actively generated cognitive processes. In everyday life, however, many forms of cognitive processes are initiated spontaneously, without an individual’s active effort and without explicit manipulation of behavioral state. Recently, there has been increased emphasis, especially in functional neuroimaging research, on spontaneous correlated activity among spatially segregated brain regions (intrinsic functional connectivity) and, more specifically, on intraindividual fluctuations of such correlated activity on various time scales (time-varying functional connectivity). In this Perspective, we propose that certain subtypes of spontaneous cognitive processes are detectable in time-varying functional connectivity measurements. We define these subtypes of spontaneous cognitive processes and review evidence of their representations in time-varying functional connectivity from studies of attentional fluctuations, memory reactivation, and effects of baseline states on subsequent perception. Moreover, we describe how these studies are critical to validating the use of neuroimaging tools (e.g., fMRI) for assessing ongoing brain network dynamics. We conclude that continued investigation of the behavioral relevance of time-varying functional connectivity will be beneficial both in the development of comprehensive neural models of cognition, and in informing on best practices for studying brain network dynamics.

## INTRODUCTION

To many neuroscientists, the question “What is your brain doing right now?” is of great interest. The answer to this question may be different at any given moment. Although some brain activity can often be attributed to overt behaviors or changes in environmental stimuli, there is growing appreciation that spontaneous processes—not attributable to specific inputs or outputs—account for a large proportion of neural variability (Raichle, 2015; Shulman, Rothman, Behar, & Hyder, 2004).

In cognitive neuroscience, focus is commonly placed on associating brain function with changes in environmental stimuli or actively generated cognitive processes. In everyday life, however, many forms of cognitive processes occur spontaneously, without an individual’s active effort and in the absence of explicit manipulation of behavioral state. For example, thoughts that are unrelated to the current sensory environment, such as involuntary memo ries, are estimated to comprise ∼30–50% of waking life (Kane et al., 2007; Killingsworth & Gilbert, 2010; Klinger & Cox, 1987) and thus likely represent a significant source of variation in ongoing brain activity (Christoff, Irving, Fox, Spreng, & Andrews-Hanna, 2016; Kucyi, 2017; Smallwood & Schooler, 2015). Thus, an alternative approach to understanding the neural basis of everyday cognition is to observe behavioral and neural phenomena as they emerge on their own—that is, the study of spontaneous cognitive processes.

Over the past decade, there has been increased emphasis, especially in functional neuroimaging research, on spontaneous correlated activity among spatially segregated nodes of the brain (functional connectivity [FC]) that can be detected during an undirected, wakeful “resting state” (Fox & Raichle, 2007). More specifically, recently there has been a strong research focus on fluctuations of such correlated activity within and between brain networks on various time scales (Calhoun, Miller, Pearlson, & Adali, 2014; Chang & Glover, 2010; Hutchison et al., 2013; Preti, Bolton, & Van De Ville, 2016) and across all pairs of brain regions (the “functional connectome”; Bullmore & Sporns, 2009; Sporns, 2010). It is now clear that the overall organization of the functional connectome is relatively stable within individuals, regardless of context (including anesthesia/unconsciousness or during different cognitive tasks) when measured on the time scale of several minutes or longer (“static FC”; Cole, Bassett, Power, Braver, & Petersen, 2014; Krienen, Yeo, & Buckner, 2014; Laumann et al., 2015; Vincent et al., 2007). However, in the emerging field of “dynamic FC” (herein referred to as time-varying FC), modeling and analysis approaches that focus on shorter time scales suggest that spontaneous network communication, although partially constrained by anatomical connectivity, can be highly variable within an individual (Cabral, Kringelbach, & Deco, 2017; Deco, Jirsa, & McIntosh, 2011; Ghosh, Rho, McIntosh, Kotter, & Jirsa, 2008; Honey, Kotter, Breakspear, & Sporns, 2007).

Several controversies remain in the field of time-varying FC. Do spontaneous temporal fluctuations of FC reflect genuine changes in neural network configurations, or do they simply reflect sampling variability and measurement noise (Handwerker, Roopchansingh, Gonzalez-Castillo, & Bandettini, 2012; Hindriks et al., 2016; Keilholz, Magnuson, Pan, Willis, & Thompson, 2013; Laumann et al., 2017; Leonardi & Van De Ville, 2015; Liegeois, Laumann, Snyder, Zhou, & Yeo, 2017)? And critically, are spontaneous cognitive processes represented in measurable reconfigurations of FC (the main focus of this Perspective)?

We begin this Perspective by proposing a definition of spontaneous cognitive processes and describe subtypes of mental and behavioral phenomena that naturally fluctuate. We then review evidence of relationships between time-varying FC and spontaneous cognitive processes from studies of attentional fluctuations, memory reactivation, and effects of baseline brain network organization on subsequent perception. We focus specifically on these areas not because they represent the only window into the cognition-FC relationship, but because multiple studies with common approaches have been conducted for each of these domains, and common findings are beginning to emerge. On the basis of the described evidence, we propose that certain subtypes of spontaneous cognitive processes are detectable in time-varying FC measurements. Moreover, we describe how these studies are critical to validating the use of neuroimaging tools (e.g., fMRI) for assessing ongoing brain network dynamics.

## WHAT ARE SPONTANEOUS COGNITIVE PROCESSES?

Spontaneous cognitive process is an umbrella term for several forms of self-generated, freely emerging thoughts, experiences, and/or sensations that are not initiated by overt, active control or by an immediate external stimulus. Following the spontaneous onset of such a process, control, intention, and other cognitive processes can become engaged. Thus, the main difference between a spontaneous and a task-evoked cognitive process is in the manner of initiation (cf. Smallwood, 2013).

All spontaneous cognitive processes are represented in the brain’s ongoing activity. However, spontaneous brain activity also reflects other intrinsic neural processes that typically do not overtly impact or reflect immediate cognition, such as maintenance of homeostasis and the integrity of anatomical connections (Raichle, 2015; Sadaghiani & Kleinschmidt, 2013). Thus, when measured over at least several minutes, overall patterns of intrinsic functional network organization are relatively insensitive to current cognition and are relatively stable within an individual over time and across contexts, with quantifiable differences across individuals (akin to a “fingerprint”; Finn et al., 2015; Laumann et al., 2015; D. Wang et al., 2015). In contrast, neural activity representing spontaneous cognitive processes is transient and state dependent. Whereas a change in cognitive state (i.e., cognitive processes in which someone is currently engaging) is always reflected in a brain state (e.g., the whole-brain pattern of FC), a change in brain state can reflect a number of factors unrelated to current cognition. Thus, a change in brain state does not implicitly suggest that a specific change in cognitive processes is occurring (Cohen, 2017; Kucyi, 2017).

Importantly, the presence of state-dependent spontaneous brain activity does not immediately imply that such activity is deviant from an individual’s functional connectome fingerprint. Indeed, individuals may have traitlike tendencies to frequently visit a given FC state during wakeful rest (Choe et al., 2017; Hutchison & Morton, 2015; Kucyi & Davis, 2014; Kucyi, Salomons, & Davis, 2013; Yang, Craddock, Margulies, Yan, & Milham, 2014). However, there may be situations in which transient changes in FC represent deviations from the fingerprint (see sections *Attention* and *Baseline Connectivity and Perceptual Variability*). Accordingly, changes in time-varying FC can also reflect changes within an individual and their day-to-day cognitive state (Shine, Koyejo, & Poldrack, 2016). State-dependent deviations from an individual’s fingerprint often occur within and between networks that are relevant for the particular cognitive process that is immediately occurring, without grossly altering other networks. This leaves the overall functional connectome pattern relatively intact (Cole et al., 2014; Geerligs, Rubinov, Cam, & Henson, 2015; Krienen et al., 2014).

Although there is acknowledgment that spontaneous cognitive processes may partially explain local neural fluctuations (Fox & Raichle, 2007) and time-varying FC (Cohen, 2017; Hutchison et al., 2013; Liegeois et al., 2017; Preti et al., 2016), there remains no consensus on a taxonomy that accurately captures the multifaceted nature of these processes. The terms “spontaneous cognition” and “spontaneous thought” have commonly been used in the literature to refer specifically to conscious, inner experiences, with some acknowledgment that there are discrete subtypes of such experiences (e.g., mind-wandering vs. intrusive/ruminative thoughts; Andrews-Hanna, Reidler, Huang, & Buckner, 2010; Buckner, Andrews-Hanna, & Schacter, 2008; Christoff et al., 2016; Giambra, 1977; Maillet & Schacter, 2016). Spontaneous cognitive processes as defined here include, but are not limited to, conscious experiences, and also include processes that are spontaneously initiated and that then subsequently occur unconsciously. We propose that there are multiple forms of spontaneous mental processes that can be described in terms of discrete categories, of which a subset are “cognitive” in nature. These spontaneous mental processes include affective state, arousal/wakefulness, hunger/thirst, sexual drive, vigilance, attention, memory (reactivation/replay), level of full consciousness, current conscious contents, and processes that mediate the onset, maintenance, and transitions of spontaneous thoughts.

**Figure 1.**
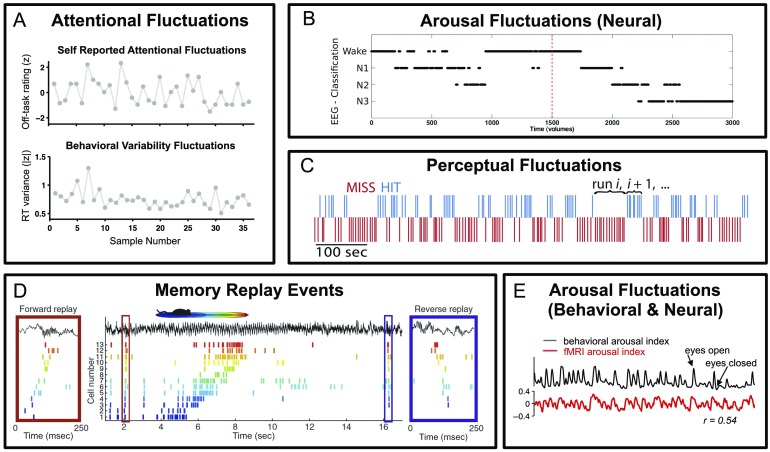
Subtypes of spontaneous cognitive processes detected in behavioral and neural fluctuations. (A) Single-subject example of attentional fluctuations during a continuous performance task, detected with intermittent self-reports of “off-task” focus (top) and with spontaneous changes in reaction time variability (bottom) (reproduced with permission from Kucyi, Esterman, Riley, & Valera, 2016). (B) Example of arousal fluctuations detected in EEG signatures of wakefulness and sleep stages (N1, N2, N3) (reproduced with permission from Haimovici, Tagliazucchi, Balenzuela, & Laufs, 2017). (C) Example of fluctuations in self-reported perception of a near-threshold stimulus across different trials (reproduced with permission from Monto et al., 2008). (D) Example of neuronal spiking activity patterns representing memory replay events that are compressed in time and may occur in forward and reverse temporal directions (reproduced with permission from Diba & Buzsaki, 2007). (E) Example of arousal fluctuations detected in behavior (spontaneous eye closures) and in a proposed fMRI neural activity marker (reproduced with permission from Chang et al., 2016).

Different spontaneous mental processes fluctuate on multiple time scales and may display unique temporal properties (Figure 1). For example, arousal/wakefulness and its impact on cognitive state typically displays a slow drift on the scale of minutes to hours (Tagliazucchi & van Someren, 2017). Spontaneous fluctuations in attention, which may be indexed by fluctuations in behavioral performance, can vary in an aperiodic fashion between focused and unfocused states on scales of seconds to 10s or 100s of seconds (Castellanos et al., 2005; Di Martino et al., 2008; Monto et al., 2008; Smallwood, McSpadden, Luus, & Schooler, 2008; Verplanck, Collier, & Cotton, 1952). In contrast, spontaneous reactivations of memories or representations of recent experience may be marked by fast transient events at the subsecond level (Diba & Buzsaki, 2007; Nadasdy, Hirase, Czurko, Csicsvari, & Buzsaki, 1999), which in turn can manifest as specific patterns in the structure of correlated activity measured over longer time scales, such as minutes (Kudrimoti, Barnes, & McNaughton, 1999; Lansink, Goltstein, Lankelma, McNaughton, & Pennartz, 2009).

Some spontaneous mental processes are interrelated with one another, such as arousal and vigilance. Others typically are relatively independent, such as attention and valence of affective state. Furthermore, some processes are composed of multiple dimensions. As an example, affect can be described on low versus high intensity or positive versus negative continuums. Moreover, differences in the content and nature of spontaneous thoughts (e.g., thoughts about the past vs. future, positive or negative) can be independent of one another and have diverging relationships with neural activity at intraindividual (Tusche, Smallwood, Bernhardt, & Singer, 2014) and interindividual levels (Karapanagiotidis, Bernhardt, Jefferies, & Smallwood, 2017; Poerio et al., 2017; Smallwood et al., 2016). Whereas some spontaneous mental processes are clearly in the “cognitive” category (e.g., attention, memory), such a classification for others is less clear (e.g., wakefulness, affective state). We focus herein on spontaneous processes that are unambiguously considered to be cognitive in nature and that have been examined in the context of time-varying FC. These include attention, memory reactivation, and perceptual processes.

During a typical experimental resting state period, and presumably during natural settings in everyday life, multiple spontaneous cognitive processes occur simultaneously. Compared with the conventional study of active cognitive processes via “task minus baseline” approaches, linking spontaneous cognitive processes to neural network dynamics presents unique empirical challenges. For example, the onset of a spontaneous cognitive process is often hidden to the observer (Smallwood, 2013). Moreover, when studying spontaneous cognitive processes in the context of time-varying FC in particular, analysis approaches including the use of network science tools (see Box 1) must be carefully chosen because of the high-dimensional, multivariate nature of time-varying connectivity data. On the basis of behavioral studies, fluctuations in physiological parameters such as pupil diameter and heart rate have been proposed to be markers of some spontaneous cognitive processes (Smallwood et al., 2004, 2011). We propose here that brain activity that more directly reflects cognitive functions may (at least in part) be captured in time-varying connectivity analyses, and could provide greater sensitivity and specificity. In the following sections, we review experimental designs and analyses that have been used in existing studies to provide evidence that multiple subtypes of spontaneous cognitive processes are represented in time-varying FC.

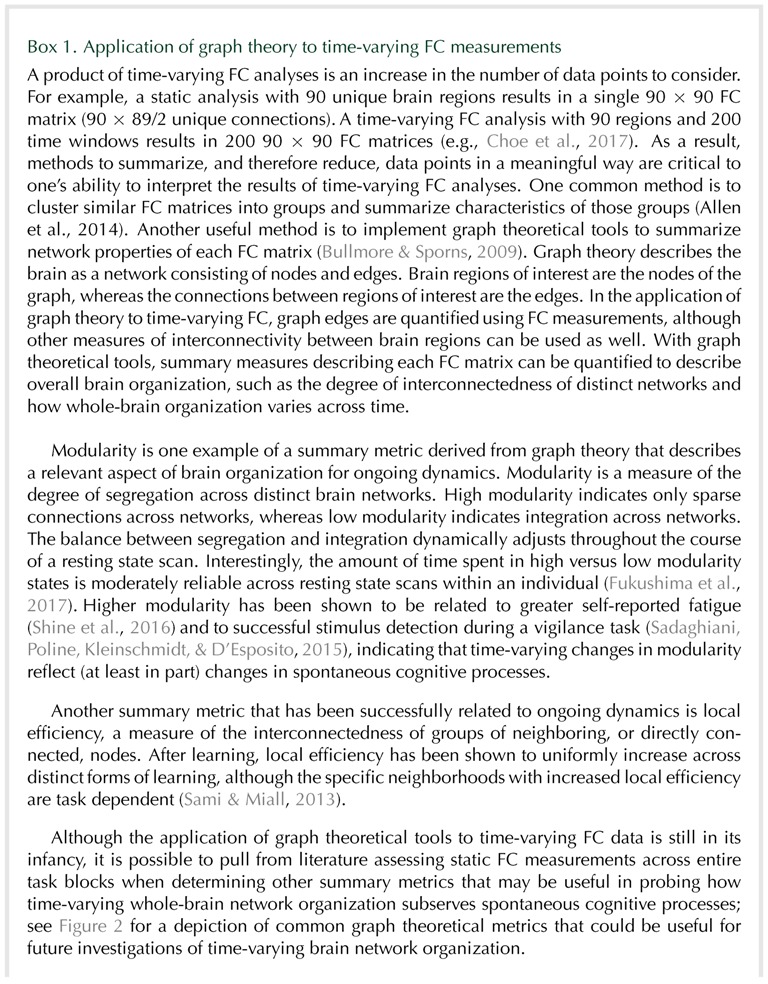

**Figure 2.**
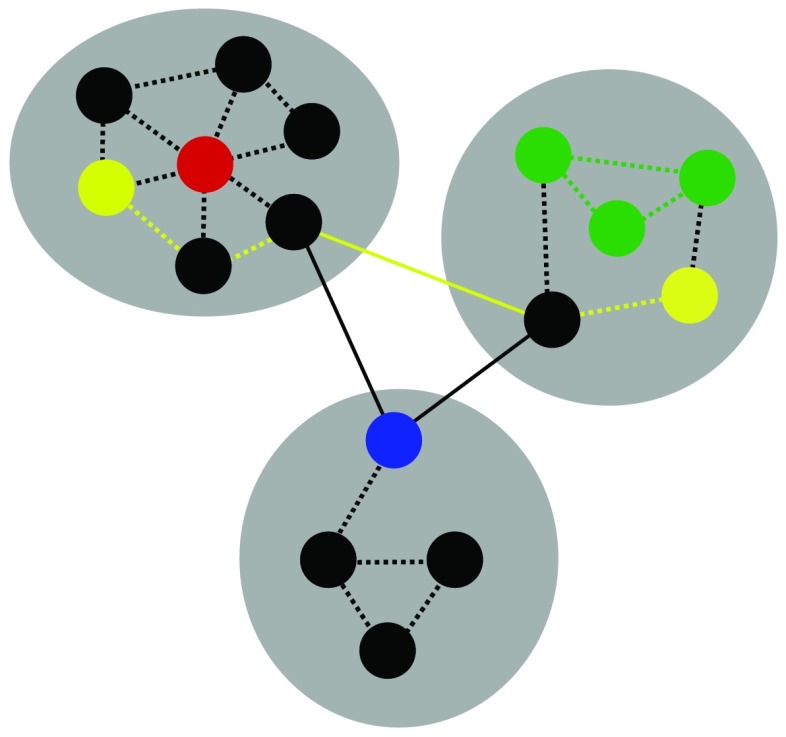
Measures of network organization. Gray ovals arenetworks of the graph, circles are nodes, solid and dashed lines are edges. Modularity is a measure of network segregation into distinct networks, with sparse connections across networks. System segregation is a measure of the relative strength of within-network connections (dashed lines) to between-network connections (solid lines). Green nodes and edges depict a cluster of nodes with high local efficiency (the efficiency of information transfer among neighboring, or directly connected, nodes). Yellow edges depict the shortest path between the two yellow nodes; the shorter the average path length across all pairs of nodes, the higher the global efficiency (the efficiency of information transfer across the entire system). The red node depicts a provincial hub (strong within-network, but weak between-network connectivity).The blue node depicts a connector hub (strong between-network, but weak within-network connectivity). Figure and caption from Cohen & D’Esposito, 2016).

### Attention

Neuroscientific investigation of attention has classically focused on aspects such as reorienting to sensory stimuli in the immediate environment (“bottom-up” or stimulus-driven attention) and to active control over the allocation of attentional resources (“top-down” or goal-driven attention; Corbetta & Shulman, 2002). However, inspired by recent discoveries of the functional importance of spontaneous activity throughout the human brain, the study of spontaneous aspects of attention has expanded (Christoff et al., 2016; Kucyi, 2017; Palva & Palva, 2011; Sonuga-Barke & Castellanos, 2007). Spontaneous attentional fluctuations include switches of focus between the present external environment and self-generated, stimulus-independent thoughts. They can also include changes of focus within each of those classes, such as a spontaneous change in the content of stimulus-independent thought. Spontaneous attentional fluctuations can be detected via behavioral performance variations or in self-reports, and both have been linked with time-varying FC. Importantly, the examinations of these “objective” and “subjective” measures are complementary to one another. Whereas behavioral fluctuations measured with response-time variability can serve to validate self-reported experiences at intra- and interindividual levels (Kucyi et al., 2013; Smallwood, 2011; Smallwood et al., 2008; Stawarczyk, Majerus, Maj, Van der Linden, & D’Argembeau, 2011), the two classes of measures also capture different aspects of attentional fluctuations and their associated neural and physiological correlates (Konishi, Brown, Battaglini, & Smallwood, 2017; Kucyi, Esterman, Riley, & Valera, 2016).

**Figure 3.**
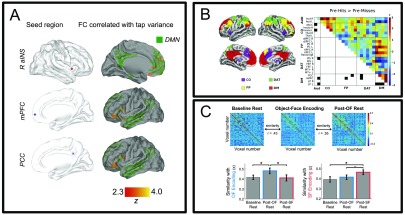
Time-varying FC correlates of spontaneous cognitive processes. (A) Attentional fluctuations, as detected in fluctuations of behavior during a prolonged period of continuous rhythmic finger tapping, are correlated with second-to-second time-varying FC of the right anterior insula (R aINS, top), medial prefrontal cortex (mPFC, middle), and posterior cingulate cortex (PCC, bottom) with regions within the default network (reproduced with permission from Kucyi et al., 2017). (B) Hippocampal FC patterns persist from encoding blocks to rest periods after encoding, suggesting that memory reactivation can be detected in minutes-long time-varying FC within the hippocampus. The similarity of resting (spontaneous) FC with FC during object-face (OF) memory encoding is greater after, compared with prior to, encoding (top). Effects are specific to the type of stimuli encoded, regardless of whether pairings are OF or scene-face (SF) (bottom) (reproduced with permission from Tambini & Davachi, 2013). (C) Baseline FC states, involving default (DM), dorsal attention (DAT), cingulo-opercular (CO) and frontoparietal (FP) networks, 22–40 s prior to auditory stimuli presented at threshold level predict whether or not stimuli will be perceived (reproduced with permission from Sadaghiani et al., 2015).

Behavioral fluctuations can be measured as variations of reaction times during continuous performance tasks that have minimal variations over time with regard to cognitive demand. Such fluctuations provide clues as to how attention naturally waxes and wanes within individuals. Spontaneous fluctuations in response-time variability have been linked with regional fMRI activity in attention-relevant brain networks, including the default network, as well as the dorsal and ventral attention networks (Esterman, Noonan, Rosenberg, & Degutis, 2013; Esterman, Rosenberg, & Noonan, 2014; Kucyi et al., 2016; Kucyi, Hove, Esterman, Hutchison, & Valera, 2017; Rosenberg, Finn, Constable, & Chun, 2015). Moreover, behavioral fluctuations have been related to time-varying FC of default and salience networks, over and above regional activation levels within these networks (Kucyi et al., 2017). Specifically, when subjects were instructed to tap their finger continuously and rhythmically over a period of several minutes, their tapping fluctuated between periods of high and low variability, with high variability periods potentially signifying attentional lapses. These continuous drifts of attention were associated with increasing time-varying FC within default network areas and increased coupling/reduced anticorrelation between salience and default network areas (Kucyi et al., 2017) (Figure 3A). A recent study in sleep-deprived subjects also identified reduced anticorrelated activity between default network regions and both salience and dorsal attention network regions as related to both spontaneous eyelid closures (assumed to reflect low arousal) and to slow response speeds during continuous task performance (C. Wang, Ong, Patanaik, Zhou, & Chee, 2016).

Importantly, behavioral performance can vary over time with or without a person’s awareness. To capture a person’s inner experience of their attentional state, self-reports can be conducted intermittently, usually with “thought probes” that are at least tens of seconds apart (Smallwood & Schooler, 2006). Thought probes can be designed to capture experiences of spontaneous attentional lapses that people were aware of either during or after the episode itself (Christoff, Gordon, Smallwood, Smith, & Schooler, 2009). Importantly, to do this successfully, the form and content of the self-reported experience must be assessed appropriately to distinguish a spontaneous attentional lapse from, for example, an intentional train of thought (Seli, Risko, & Smilek, 2016). Although they do not provide the same temporal resolution as behavioral performance fluctuations, self-reports can provide critical information about how an attentional fluctuation was experienced. For example, attention may fluctuate because of spontaneous mind wandering or because of an experienced external distraction (Smallwood & Schooler, 2015; Stawarczyk et al., 2011).

Initial neuroimaging studies reported associations of self-reported attentional fluctuations, as assessed using online thought probes, with regional activity changes in default and attention-relevant brain networks (Christoff et al., 2009; Vanhaudenhuyse et al., 2011). Additionally, recent studies using time-varying FC analyses of temporal windows prior to thought probes (ranging from 20 to 40 seconds) have revealed that subjective, spontaneous fluctuations in attention away from both pain (self-reported mind wandering; Kucyi et al., 2013) and task performance (self-reported “off-task” thought; Mittner et al., 2014) are associated with intra- and internetwork connectivity of the default network. Additionally, FC correlates of intraindividual variations in self-reported attentional states have been observed on the time scale of minutes. At the within-network level, default network FC variability has been associated with intensity of mind wandering (Kucyi & Davis, 2014). On the whole-brain network level, flexible interareal dynamics have been associated with heightened attention (Shine et al., 2016).

Although the studies summarized herein largely required the introduction of a task or the interruption of ongoing experience, nevertheless they provide a window into the types of attentional fluctuations (e.g., variations in mind-wandering intensity) that occur spontaneously and that are likely to be represented in time-varying FC during wakeful rest. We therefore propose that spontaneous FC fluctuations within and between particular networks (e.g., default and salience networks), and in whole-brain network-level properties (e.g., flexible interareal dynamics), reflect the waxing and waning of attention that naturally occurs at rest. Future studies to test this hypothesis could involve the comparison of neural activity that is associated with self-report and behavioral measures of attention versus independent neural activity measured at rest within the same subjects. Combined with putative covert measures of attention (cf. C. Wang et al., 2016), such an approach could be fruitful in potentially enabling objective tracking of attentional states in time-varying FC patterns.

### Baseline Connectivity and Perceptual Variability

Similar to the above-described fluctuations in attention, and likely under the influence thereof, perception is often highly variable within an individual even when stimuli and external conditions are held constant (Gilden, 2001; Kleinschmidt, Sterzer, & Rees, 2012). The investigation of perceptual variability enables understanding of how spontaneous cognitive processes that influence the internal momentary state of the brain modulate awareness of the external world. Beyond contributing to an understanding of the cognitive significance of connectome dynamics, the investigation of perceptual variability also adds to the validation of the neural origin of time-varying FC (see section *Validating Time-Varying Functional Connectivity: Challenges and Opportunities*).

Spontaneous fluctuations in perception of external sensory stimuli correlate with variability in evoked neural responses (Bernasconi et al., 2011; Pessoa & Padmala, 2005; Ratcliff, Philiastides, & Sajda, 2009; Ress, Backus, & Heeger, 2000). Evoked response variability in turn has been linked to spontaneous fluctuations in regional amplitude of baseline or ongoing activity in both task-relevant sensory brain regions (Hesselmann, Kell, Eger, & Kleinschmidt, 2008; Hesselmann, Kell, & Kleinschmidt, 2008; Sadaghiani, Hesselmann, & Kleinschmidt, 2009), as well as in large-scale higher order brain networks (Boly et al., 2007; Coste, Sadaghiani, Friston, & Kleinschmidt, 2011; Sadaghiani et al., 2009). As discussed below, more recent investigations have moved beyond such univariate analyses of spontaneous activity, suggesting that the correlation of baseline amplitude fluctuations across distributed regions, which emerges as spontaneous time-varying FC, is related to variability in perception.

Studying the perceptual consequences of connectome dynamics inherently requires a task. Indeed, functional connectome dynamics as observed with fMRI have been investigated during task performance (e.g., Bassett et al., 2011; Davison et al., 2015; Ekman, Derrfuss, Tittgemeyer, & Fiebach, 2012; Shine et al., 2016; Simony et al., 2016). However, such studies do not directly speak to the significance of spontaneous brain activity because the inherently slow hemodynamic responses evoked by external task events generally overlap in time with ongoing intrinsic activity. Any attempt to regress out evoked activity (e.g., Gonzalez-Castillo et al., 2015) may be incomplete because of trial-to-trial variability of the task-evoked brain responses. Although this very variability might emerge from an interaction of evoked activity and spontaneous changes in background activity, evoked and spontaneous processes cannot be disentangled with subtraction or regression methods because of their potential nonlinear interaction (He, 2013; Hesselmann, Kell, Eger, & Kleinschmidt, 2008). Thus, although these task investigations provide important information about task-dependent FC, spontaneous changes in the intrinsic functional connectome may remain nondissociable from task-evoked coactivation. To understand how spontaneous functional connectome dynamics may result in fluctuations of task-evoked neural processes, we first must study the former and the latter in a dissociable manner. Therefore, the central challenge in studying the impact of spontaneous FC shifts on perceptual variability is the difficulty in dissociating them from task-evoked coactivation during tasks.

To unequivocally attribute changes in perception to spontaneous connectome state changes, some researchers have turned to studying FC during prestimulus time periods. A shared feature of these studies is the use of stimuli that are identical or directly comparable from trial to trial, yet cause different perceptual outcomes each time, such as threshold-level or ambiguous stimuli prominent in classical evoked studies of perceptual awareness (Kleinschmidt et al., 2012). The following examples focus on fMRI because of its spatial precision that enables the mapping of functional connectome states, although electrophysiological and magnetoencephalography studies similarly tap into the behavioral impact of large-scale prestimulus connectivity at times scales of canonical oscillatory frequencies (Weisz et al., 2014). A study of threshold pain perception found that prestimulus FC between the anterior insula and brainstem across the 3 s prior to the somatosensory stimulus was stronger when stimuli were rated as painful. Variations in FC explained variability in ratings both within and across subjects (Ploner, Lee, Wiech, Bingel, & Tracey, 2010). In another study, subjects were asked to detect a sparse and inconspicuous stimulus change as an assessment of ongoing vigilance. Analyses investigated the relation between the default network and “task-positive” control regions typically activated in response to external demands (Thompson et al., 2013). It was found that detection success was best when the default network was least correlated with the control regions in the several seconds before the stimulus change and extending through the evoked hemodynamic response period. Another study of vigilance fluctuations (auditory threshold detection) with very long intertrial intervals unaffected by evoked brain responses (22–40 s) applied whole-brain connectomics (graph theory, Box 1) and classification procedures during prestimulus time to establish the predictive power of spontaneous connectome dynamics (Sadaghiani, Poline, Kleinschmidt, & D’Esposito, 2015) (Figure 3B). Classification of single-trial large-scale network correlations predicted hits versus misses. Graph investigations revealed that the functional connectome alternated between states of high and low modularity (i.e., states of more or less segregated neurocognitive networks, respectively), with higher modularity scores preceding successful stimulus detection. Like whole-brain connectome investigations applied to entire continuous task periods (Shine et al., 2016) or to a task-free resting state (Betzel, Fukushima, He, Zuo, & Sporns, 2016), this study highlights the functional importance of modularity of brain states, which describes network organization across a segregation-integration axis.

Taken together, studies of baseline activity have highlighted that detectable, spontaneous reconfigurations of FC are predictive of upcoming behavioral performance. Critically, prestimulus FC explains perceptual variability over and above regional (univariate) activity (Sadaghiani et al., 2015). Similar within- and between-network interactions are likely to be present in time-varying FC during wakeful rest. Although behavioral output measures are absent in a resting state experiment, such fluctuations likely reflect and impact an individual’s unobserved cognitive state.

### Memory Reactivation

Another spontaneous cognitive process that has been investigated using time-varying FC is memory reactivation, or the recurrence of representations of past experience after events are initially encoded into memory. Although overt or explicit retrieval of information from memory is associated with the reactivation of representations present during the encoding of events (Ritchey, Wing, LaBar, & Cabeza, 2013), memory reactivation can also occur in a spontaneous manner after learning. Spontaneous memory reactivation, in conjunction with large-scale network-level interactions (e.g., hippocampal-cortical interactions), are major mechanisms thought to support long-term memory stabilization. By recording the activity of neural ensembles before, during, and after behavioral tasks, animal studies have demonstrated an increase in the spontaneous emergence of task-evoked sequences of firing from pre- to posttask periods (e.g., Ji & Wilson, 2007), and that features of spontaneous reactivation are related to subsequent memory (Csicsvari & Dupret, 2014). This spontaneous reactivation tends to occur during “off-line” time periods, such as awake rest periods or sleep, when the hippocampal local field potential is dominated by brief (∼100 ms) sharp-wave ripple (SWR) events (Buzsaki, Horvath, Urioste, Hetke, & Wise, 1992; Buzsaki, Leung, & Vanderwolf, 1983; Diba & Buzsaki, 2007).

Inspired by ensemble-level reactivation, time-varying FC approaches have been used to query the existence of spontaneous reactivation via resting state fMRI. Although SWRs and reactivation events occur at a faster time scale than the BOLD response, individual hippocampal ripple events are associated with transient but robust and widespread BOLD signal changes (Logothetis et al., 2012), particularly in the default network (Kaplan et al., 2016). Moreover, the occurrence of repeated reactivation events can systematically alter the spontaneous correlation structure of firing patterns, resulting in a persistence of connectivity patterns induced during a prior task at a longer time scale of minutes (Kudrimoti et al., 1999; Lansink et al., 2009). Researchers have investigated reactivation after the cessation of learning in fMRI by examining transient pattern reactivation at individual time points (de Voogd, Fernandez, & Hermans, 2016; Deuker et al., 2013; Gruber, Ritchey, Wang, Doss, & Ranganath, 2016; Schlichting & Preston, 2014; Staresina, Alink, Kriegeskorte, & Henson, 2013) or by analyzing the persistence of learning-related FC patterns over the course of minutes (Hermans et al., 2017; Tambini & Davachi, 2013). Both approaches have shown that activity patterns present during learning more closely match spontaneous patterns present during rest after learning compared with before learning, providing evidence consistent with the underlying reactivation of recently acquired memories (de Voogd et al., 2016; Gruber et al., 2016; Tambini & Davachi, 2013) (Figure 3C). Importantly, the persistence of learning-related patterns during postlearning periods is positively related to memory performance when it is tested hours later, suggesting that experience-dependent changes in spontaneous activity are related to the stabilization of memory for recent experience (de Voogd et al., 2016; Deuker et al., 2013; Gruber et al., 2016; Schlichting & Preston, 2014; Staresina et al., 2013). The persistence of learning-related patterns occurs in medial temporal lobe regions, including the hippocampus (Gruber et al., 2016; Hermans et al., 2017; Tambini & Davachi, 2013), amygdala (Hermans et al., 2017), and entorhinal cortex (Staresina et al., 2013), as well as in retrosplenial cortex (Staresina et al., 2013) and ventral temporal cortex (de Voogd et al., 2016; Schlichting & Preston, 2014). These findings indicate that neural signatures consistent with underlying spontaneous memory-related pattern reactivation can be observed at multiple time scales across multiple brain regions.

Beyond measuring the reemergence of learning-related patterns within individual brain regions, other studies have examined changes in levels of FC across sets of regions engaged during learning. For example, experience-dependent changes in hippocampal-cortical FC from pre- to postencoding periods tracks overall levels of later associative memory across different encoding experiences and predicts memory across participants (Murty, Tompary, Adcock, & Davachi, 2016; Tambini, Ketz, & Davachi, 2010). Furthermore, hippocampal-cortical FC has been related to the integration of recently learned information with information encountered minutes later (Schlichting & Preston, 2014). In addition to paradigms focusing on memory supported by the hippocampal memory system, experience-dependent changes in resting FC occur after motor learning (Albert, Robertson, & Miall, 2009; Gregory, Robertson, Manoach, & Stickgold, 2016; Sami, Robertson, & Miall, 2014), visual perceptual training (Lewis, Baldassarre, Committeri, Romani, & Corbetta, 2009; Mackey, Miller Singley, & Bunge, 2013), and neurofeedback (Harmelech, Preminger, Wertman, & Malach, 2013). Collectively, these findings indicate that a broad set of experiences can bias the spontaneous correlation structure observed during rest.

Although it is clear that spontaneous FC patterns change over time in a manner consistent with underlying reactivation and interactions that support later memory, it is currently unknown whether these BOLD measures directly reflect the underlying replay of neural ensembles. Replay events can be observed with high fidelity in neurophysiological recordings; however, the detection of reactivation events via fMRI in the absence of validating measures (as in Logothetis et al., 2012) is not nearly as precise, which is likely due to the sensitivity of BOLD to nonneural signals as well as the reduced spatial resolution of fMRI. It is thus possible that changes in FC observed in prior studies may reflect the presence of multiple memory mechanisms, such as both underlying reactivation and Hebbian plasticity (Harmelech & Malach, 2013), as well as brain activity not directly related to memory for recent experiences. Neural signatures consistent with spontaneous reactivation have been observed during rest periods, sleep, and subsequent task performance (Bergmann, Rijpkema, Fernandez, & Kessels, 2012; Tompary, Duncan, & Davachi, 2015). However, the precise principles underlying when postencoding memory reactivation may occur, as well as whether spontaneous reactivation indicates the existence of conscious thoughts regarding recently encountered information (Dewar, Cowan, & Sala, 2007), are currently unclear.

Importantly, memory reactivation is thought to occur frequently in daily life after learning of novel information. Thus, evidence from the above-described studies implies that time-varying FC during typical wakeful rest is likely to be affected by spontaneously occurring memory reactivation, at least in some networks. Currently, evidence for reactivation has been primarily examined within specific brain regions or in interactions across particular sets of brain regions (e.g., hippocampal-cortical interactions). Given that hippocampal SWR events are associated with spatially widespread changes in BOLD activity, an important next step is to examine how memory-related changes in spontaneous activity manifest at the level of whole-brain FC patterns and potentially influence large-scale graph theoretical properties of whole-brain networks (see Sami & Miall, 2013, Box 1).

## VALIDATING TIME-VARYING FUNCTIONAL CONNECTIVITY: CHALLENGES AND OPPORTUNITIES

One of the greatest challenges in the field of time-varying FC is validation. Early studies in the field demonstrated the difficulty of disentangling apparent fluctuations in FC that arise from the heavily autocorrelated nature of the preprocessed BOLD signal from true changes in FC that reflect the time-varying organization of brain activity (Handwerker et al., 2012; Keilholz, Magnuson, Pan, Willis, & Thompson, 2013). These issues have continued to haunt the field as it has grown.For example, sliding window correlation, one of the simplest and most popular techniques for analysis, has been repeatedly shown to provide a suboptimal representation of underlying network dynamics (Hindriks et al., 2016; Laumann et al., 2017; Leonardi & Van De Ville, 2015; Shakil, Lee, & Keilholz, 2016). New model-based methods to assess dynamic changes in FC have been developed that increase within-subject reliability and are better able to differentiate data that is known to be stationary from that which is purportedly dynamic (Choe et al., 2017). Part of the difficulty, however, in performing the correct statistical analysis of time-varying connectivity data is that the appropriate null model is unknown. Because no other existing modality can obtain information about activity throughout the human brain with the spatial and temporal resolution of fMRI, researchers are in the precarious position of trying to use time-varying FC to learn about the macroscopic organization of the brain while trying to use what they know about macroscopic brain activity to determine the appropriate models for analysis. Without external means of validating the findings or assessing the relative merits of new methods, it would be difficult for time-varying FC analysis of resting state fMRI data to move forward.

Fortunately, possibilities for external validation exist. At a group level, dynamic information differentiates some cohorts of patients from healthy controls (Jones et al., 2012; Kaiser et al., 2016; Li et al., 2014; Rashid, Damaraju, Pearlson, & Calhoun, 2014) and relates to individual variability in cognitive traits (Cheng et al., 2017; Kucyi & Davis, 2014; Kucyi et al., 2013; Shen et al., 2015; Yang et al., 2014), including in situations in which static FC measurements do not correlate with or relate independently with such traits (Cheng et al., 2017; Kucyi & Davis, 2014; Kucyi et al., 2013). This confirms that time-varying FC analysis provides additional useful information over and above static FC measurements. However, ideally validation would be performed on a finer scale, assessing sensitivity to intraindividual changes. This can be accomplished by simultaneous measurement of another neural signal that is undeniably sensitive to changes in neuronal activity (e.g., EEG) or a proxy behavioral measure of the brain’s varying activity (e.g., reaction time). EEG can be readily combined with resting state fMRI to provide an external estimate of the state of the brain and has been used to show that large-scale changes in the BOLD signal are linked to changes in the pattern of neural activity, particularly those that may reflect arousal levels (Allen, Damaraju, Eichele, Wu, & Calhoun, 2017; Chang, Liu, Chen, Liu, & Duyn, 2013; Grooms et al., 2017; Hiltunen et al., 2014; Tagliazucchi, von Wegner, Morzelewski, Brodbeck, & Laufs, 2012). Because the spatial resolution of EEG is rather poor and sensitivity is limited to the cortex, it is more appropriate for the validation of large-scale changes than for localized dynamics. Invasive recordings in animals have shown that the fluctuations in the BOLD signal correlate to local field potentials from the same site (Pan et al., 2011; Pan, Thompson, Magnuson, Jaeger, & Keilholz, 2013; Shmuel & Leopold, 2008), and that BOLD sliding window correlation reflects changes in local field potential correlation, particularly in the theta, beta, and gamma bands (Thompson et al., 2013). Human intracranial EEG studies, which have revealed frequency-specific FC patterns on the time scale of minutes that resemble BOLD FC (Foster, Rangarajan, Shirer, & Parvizi, 2015; Hacker, Snyder, Pahwa, Corbetta, & Leuthardt, 2017; He, Snyder, Zempel, Smyth, & Raichle, 2008; Keller et al., 2013; Nir et al., 2008) may also be useful in the future for validating and revealing mechanistic details about time-varying BOLD FC.

Instead of measuring brain activity directly, behavioral output, as summarized in the above sections, can be used as a proxy for alterations in neural activity. One of the drawbacks of behavioral outputs for validation of time-varying FC is that even when tasks with low cognitive load are used, the subject is by definition no longer in the “resting state.” However, as discussed above, if the resting state is considered to be undirected cognition, it is likely to contain many of the same processes that can be assessed using tasks (e.g., attentional fluctuations, memory recall, imagery, etc.), which implies that some of the same changes should be observed. Another caveat is that behavioral outputs are informative about FC within and between networks involved in the task but not about the specific behavior of interest. For example, in a task in which the subject determines whether the current stimulus has been presented before, the accuracy of the answer and the time taken for a decision may be linked to different aspects of time-varying FC. Moreover, behavioral outputs can only be used to validate dynamics related to areas involved in the task, leaving time-varying FC in much of the rest of the brain difficult to interpret. Task performance and behavior may also be linked to various levels of motion, a potentially confounding factor for time-varying FC (Siegel et al., 2017).

It is clear that care is needed in validating time-varying FC analyses of resting state fMRI. One of the factors that is sometimes overlooked is that the dynamics expected from the brain range widely across spatial and temporal scales. Indeed, there is evidence that resting state 1fMRI contains information from the localized scale of columnar or areal activity to the global scale of whole-brain fluctuations, with multiple independent neurophysiological sources (Hyde & Li, 2014; Keilholz, 2014; Keilholz, Pan, Billings, Nezafati, & Shakil, 2017; Raemaekers et al., 2014; Thompson, Pan, Billings, Grooms, Shakil, Jaeger, & Keilholz, 2014; Thompson, Pan, & Keilholz, 2015; Thompson, Pan, Magnuson, Jaeger, & Keilholz, 2014; Thompson et al., 2016; Wong, DeYoung, & Liu, 2016). Thus, dynamic patterns at one spatial and temporal scale may reflect fluctuations in vigilance (Chang et al., 2016), whereas another scale may be better suited to the changes associated with other spontaneous cognitive processes such as memory reactivations. As better models of the brain’s macroscale organization are developed, more sensitive methods for specifically detecting the changes that are of interest are likely to emerge.

## CONCLUSION

The evidence described herein suggests that certain subtypes of spontaneous cognitive processes—those that are likely to occur frequently during any given session of wakeful rest—are detectable in specific patterns of time-varying FC. Studies of attentional fluctuations, memory reactivation, and the relationship between baseline brain activity and variation in perception have each shown that spontaneous and transient changes in BOLD FC are relevant to behavior and experience. By showing that interregional interactions contain behaviorally relevant information over and above regional activation levels, these studies have critically contributed to the validation of time-varying FC measures. Although more research that replicates and extends the described findings to other cognitive processes that can occur spontaneously would improve confidence, the existing body of evidence should convince researchers of the importance and tractability, with currently existing neural recording techniques, of the question of how spontaneous cognitive processes are represented in time-varying FC. Future research should build on the body of research described here as well as focus on classes of spontaneous cognitive processes that we omitted from our discussion. The continued study of spontaneous cognitive processes is not only necessary for addressing the question of “What is your brain doing right now,” but also for the development of comprehensive neural models of cognition and for informing on best practices for studying brain network dynamics.

## ACKNOWLEDGMENTS

We thank Jonathan Smallwood for thoughtful comments on a draft of this manuscript.

## AUTHOR CONTRIBUTIONS

Aaron Kucyi, Arielle Tambini, Sepideh Sadaghiani, Shella Keilholz, and Jessica R. Cohen: Conceptualization; Writing.

## FUNDING INFORMATION

Aaron Kucyi, Canadian Institutes of Health Research (http://dx.doi.org/10.13039/501100000024) Arielle Tambini, National Institutes of Health (http://dx.doi.org/10.13039/100000002), Award ID: NIH F32 MH106280. Shella Keilholz, National Institutes of Health (http://dx.doi.org/10.13039/100000002), Award ID: R01MH111416. Shella Keilholz, National Institutes of Health (http://dx.doi.org/10.13039/100000002), Award ID: R01NS078095. Shella Keilholz, National Science Foundation (http://dx.doi.org/10.13039/100000001), Award ID: BCS INSPIRE 1533260. Jessica R. Cohen, National Institute of Mental Health (http://dx.doi.org/10.13039/100000025), Award ID: R00MH102349.

## TECHNICAL TERMS

Spontaneous cognitive processes: A freely emerging thought, experience, and/or sensation that is not initiated by active control or by an immediate external stimulus.
Functional connectivity: Statistical dependence (e.g., correlation) between the activity levels within two brain regions.
Functional connectome: A description of the functional connectivity among all pairs of brain regions.
Time-varying functional connectivity: Change in functional connectivity across independent or semioverlapping time windows within an individual.
Memory reactivation: Recurrence of neural representations of past experience after events are initially encoded into memory.
Intrinsic neural process: Spontaneous brain activity that cannot be attributed to active control or to an immediate external stimulus.
Spontaneous thought: A type of spontaneous cognitive process that involves conscious awareness (e.g., mind-wandering, rumination).
Spontaneous mental process: A freely emerging neural process that is reflected in a behavior or experience (a spontaneous cognitive process is a subtype).
Perceptual variability: The notion that the same sensory stimulus can have different perceptual consequences at different moments.
Evoked neural response: Increase in brain activity or change in functional connectivity initiated by sensory stimuli or external change in task set/cognitive demand.

## References

[bib1] Albert N. B., Robertson E. M., & Miall R. C. (2009). The resting human brain and motor learning. Current Biology, 19(12), 1023–1027. 10.1016/j.cub.2009.04.028 19427210 PMC2701987

[bib2] Allen E. A., Damaraju E., Eichele T., Wu L., & Calhoun V. D. (2017). EEG signatures of dynamic functional network connectivity states. Brain Topography, 31, 101–116. 10.1007/s10548-017-0546-2 28229308 PMC5568463

[bib3] Allen E. A., Damaraju E., Plis S. M., Erhardt E. B., Eichele T., & Calhoun V. D. (2014). Tracking whole-brain connectivity dynamics in the resting state. Cerebral Cortex, 24(3), 663–676. 10.1093/cercor/bhs352 23146964 PMC3920766

[bib4] Andrews-Hanna J. R., Reidler J. S., Huang C., & Buckner R. L. (2010). Evidence for the default network’s role in spontaneous cognition. Journal of Neurophysiology, 104(1), 322–335. 10.1152/jn.00830.2009 20463201 PMC2904225

[bib5] Bassett D. S., Wymbs N. F., Porter M. A., Mucha P. J., Carlson J. M., & Grafton S. T. (2011). Dynamic reconfiguration of human brain networks during learning. Proceedings of the National Academy of Sciences of the United States of America, 108(18), 7641–7646. 10.1073/pnas.1018985108 21502525 PMC3088578

[bib6] Bergmann H. C., Rijpkema M., Fernandez G., & Kessels R. P. (2012). Distinct neural correlates of associative working memory and long-term memory encoding in the medial temporal lobe. NeuroImage, 63(2), 989–997. 10.1016/j.neuroimage.2012.03.047 22484305

[bib7] Bernasconi F., De Lucia M., Tzovara A., Manuel A. L., Murray M. M., & Spierer L. (2011). Noise in brain activity engenders perception and influences discrimination sensitivity. Journal of Neuroscience, 31(49), 17971–17981. 10.1523/JNEUROSCI.3715-11.2011 22159111 PMC6634160

[bib8] Betzel R. F., Fukushima M., He Y., Zuo X. N., & Sporns O. (2016). Dynamic fluctuations coincide with periods of high and low modularity in resting-state functional brain networks. NeuroImage, 127, 287–297. 10.1016/j.neuroimage.2015.12.001 26687667 PMC4755785

[bib9] Boly M., Balteau E., Schnakers C., Degueldre C., Moonen G., Luxen A., … Laureys S. (2007). Baseline brain activity fluctuations predict somatosensory perception in humans. Proceedings of the National Academy of Sciences of the United States of America, 104(29), 12187–12192. 17616583 10.1073/pnas.0611404104 PMC1924544

[bib10] Buckner R. L., Andrews-Hanna J. R., & Schacter D. L. (2008). The brain’s default network: Anatomy, function, and relevance to disease. Annals of the New York Academy of Sciences, 1124, 1–38. 10.1196/annals.1440.011 18400922

[bib11] Bullmore E., & Sporns O. (2009). Complex brain networks: Graph theoretical analysis of structural and functional systems. Nature Reviews Neuroscience, 10(3), 186–198. 10.1038/nrn2575 19190637

[bib12] Buzsaki G., Horvath Z., Urioste R., Hetke J., & Wise K. (1992). High-frequency network oscillation in the hippocampus. Science, 256(5059), 1025–1027. 1589772 10.1126/science.1589772

[bib13] Buzsaki G., Leung L. W., & Vanderwolf C. H. (1983). Cellular bases of hippocampal EEG in the behaving rat. Brain Research, 287(2), 139–171. 6357356 10.1016/0165-0173(83)90037-1

[bib14] Cabral J., Kringelbach M. L., & Deco G. (2017). Functional connectivity dynamically evolves on multiple time-scales over a static structural connectome: Models and mechanisms. NeuroImage, 160, 84–96. 10.1016/j.neuroimage.2017.03.045 28343985

[bib15] Calhoun V. D., Miller R., Pearlson G., & Adali T. (2014). The chronnectome: Time-varying connectivity networks as the next frontier in fMRI data discovery. Neuron, 84(2), 262–274. 10.1016/j.neuron.2014.10.015 25374354 PMC4372723

[bib16] Castellanos F. X., Sonuga-Barke E. J., Scheres A., Di Martino A., Hyde C., & Walters J. R. (2005). Varieties of attention-deficit/hyperactivity disorder-related intra-individual variability. Biological Psychiatry, 57(11), 1416–1423. 10.1016/j.biopsych.2004.12.005 15950016 PMC1236991

[bib17] Chang C., & Glover G. H. (2010). Time-frequency dynamics of resting-state brain connectivity measured with fMRI. NeuroImage, 50(1), 81–98. 10.1016/j.neuroimage.2009.12.011 20006716 PMC2827259

[bib18] Chang C., Leopold D. A., Scholvinck M. L., Mandelkow H., Picchioni D., Liu X., … Duyn J. H. (2016). Tracking brain arousal fluctuations with fMRI. Proceedings of the National Academy of Sciences of the United States of America, 113(16), 4518–4523. 10.1073/pnas.1520613113 27051064 PMC4843437

[bib19] Chang C., Liu Z., Chen M. C., Liu X., & Duyn J. H. (2013). EEG correlates of time-varying BOLD functional connectivity. NeuroImage, 72, 227–236. 10.1016/j.neuroimage.2013.01.049 23376790 PMC3602157

[bib20] Cheng J. C., Bosma R. L., Hemington K. S., Kucyi A., Lindquist M. A., & Davis K. D. (2017). Slow-5 dynamic functional connectivity reflects the capacity to sustain cognitive performance during pain. NeuroImage, 157, 61–68. 10.1016/j.neuroimage.2017.06.005 28583880

[bib21] Choe A. S., Nebel M. B., Barber A. D., Cohen J. R., Xu Y., Pekar J. J., … Lindquist M. A. (2017). Comparing test-retest reliability of dynamic functional connectivity methods. NeuroImage, 158, 155–175. 10.1016/j.neuroimage.2017.07.005 28687517 PMC5614828

[bib22] Christoff K., Gordon A. M., Smallwood J., Smith R., & Schooler J. W. (2009). Experience sampling during fMRI reveals default network and executive system contributions to mind wandering. Proceedings of the National Academy of Sciences of the United States of America, 106(21), 8719–8724. 19433790 10.1073/pnas.0900234106 PMC2689035

[bib23] Christoff K., Irving Z. C., Fox K. C., Spreng R. N., & Andrews-Hanna J. R. (2016). Mind-wandering as spontaneous thought: A dynamic framework. Nature Reviews Neuroscience, 17(11), 718–731. 10.1038/nrn.2016.113 27654862

[bib24] Cohen J. R. (2017). The behavioral and cognitive relevance of time-varying, dynamic changes in functional connectivity. NeuroImage. 10.1016/j.neuroimage.2017.09.036 PMC6056319 28942061

[bib25] Cohen J. R., & D’Esposito M. (2016). The segregation and integration of distinct brain networks and their relationship to cognition. Journal of Neuroscience, 36(48), 12083–12094. 10.1523/JNEUROSCI.2965-15.2016 27903719 PMC5148214

[bib26] Cole M. W., Bassett D. S., Power J. D., Braver T. S., & Petersen S. E. (2014). Intrinsic and task-evoked network architectures of the human brain. Neuron, 83(1), 238–251. 10.1016/j.neuron.2014.05.014 24991964 PMC4082806

[bib27] Corbetta M., & Shulman G. L. (2002). Control of goal-directed and stimulus-driven attention in the brain. Nature Reviews Neuroscience, 3(3), 201–215. 11994752 10.1038/nrn755

[bib28] Coste C. P., Sadaghiani S., Friston K. J., & Kleinschmidt A. (2011). Ongoing brain activity fluctuations directly account for intertrial and indirectly for intersubject variability in Stroop task performance. Cerebral Cortex, 21(11), 2612–2619. 10.1093/cercor/bhr050 21471558

[bib29] Csicsvari J., & Dupret D. (2014). Sharp wave/ripple network oscillations and learning-associated hippocampal maps. Philosophical Transactions of the Royal Society of London, 369(1635), 20120528 10.1098/rstb.2012.0528 24366138 PMC3866448

[bib30] Davison E. N., Schlesinger K. J., Bassett D. S., Lynall M. E., Miller M. B., Grafton S. T., & Carlson J. M. (2015). Brain network adaptability across task states. PLoS Computational Biology, 11(1), e1004029 10.1371/journal.pcbi.1004029 25569227 PMC4287347

[bib31] de Voogd L. D., Fernandez G., & Hermans E. J. (2016). Awake reactivation of emotional memory traces through hippocampal-neocortical interactions. NeuroImage, 134, 563–572. 10.1016/j.neuroimage.2016.04.026 27095308

[bib32] Deco G., Jirsa V. K., & McIntosh A. R. (2011). Emerging concepts for the dynamical organization of resting-state activity in the brain. Nature Reviews Neuroscience, 12(1), 43–56. 21170073 10.1038/nrn2961

[bib33] Deuker L., Olligs J., Fell J., Kranz T. A., Mormann F., Montag C., … Axmacher N. (2013). Memory consolidation by replay of stimulus-specific neural activity. Journal of Neuroscience, 33(49), 19373–19383. 10.1523/JNEUROSCI.0414-13.2013 24305832 PMC6618788

[bib34] Dewar M. T., Cowan N., & Sala S. D. (2007). Forgetting due to retroactive interference: A fusion of Muller and Pilzecker’s (1900) early insights into everyday forgetting and recent research on anterograde amnesia. Cortex, 43(5), 616–634. 17715797 10.1016/s0010-9452(08)70492-1 PMC2644330

[bib35] Di Martino A., Ghaffari M., Curchack J., Reiss P., Hyde C., Vannucci M., … Castellanos F. X. (2008). Decomposing intra-subject variability in children with attention-deficit/hyperactivity disorder. Biological Psychiatry, 64(7), 607–614. 10.1016/j.biopsych.2008.03.008 18423424 PMC2707839

[bib36] Diba K., & Buzsaki G. (2007). Forward and reverse hippocampal place-cell sequences during ripples. Nature Neuroscience, 10(10), 1241–1242. 10.1038/nn1961 17828259 PMC2039924

[bib37] Ekman M., Derrfuss J., Tittgemeyer M., & Fiebach C. J. (2012). Predicting errors from reconfiguration patterns in human brain networks. Proceedings of the National Academy of Sciences of the United States of America, 109(41), 16714–16719. 10.1073/pnas.1207523109 23012417 PMC3478635

[bib38] Esterman M., Noonan S. K., Rosenberg M., & Degutis J. (2013). In the zone or zoning out? Tracking behavioral and neural fluctuations during sustained attention. Cerebral Cortex, 23(1), 2712–2723. 10.1093/cercor/bhs261 22941724

[bib39] Esterman M., Rosenberg M. D., & Noonan S. K. (2014). Intrinsic fluctuations in sustained attention and distractor processing. Journal of Neuroscience, 34(5), 1724–1730. 10.1523/JNEUROSCI.2658-13.2014 24478354 PMC6827583

[bib40] Finn E. S., Shen X., Scheinost D., Rosenberg M. D., Huang J., Chun M. M., … Constable R. T. (2015). Functional connectome fingerprinting: Identifying individuals using patterns of brain connectivity. Nature Neuroscience, 18(11), 1664–1671. 10.1038/nn.4135 26457551 PMC5008686

[bib41] Foster B. L., Rangarajan V., Shirer W. R., & Parvizi J. (2015). Intrinsic and task-dependent coupling of neuronal population activity in human parietal cortex. Neuron, 86(2), 578–590. 10.1016/j.neuron.2015.03.018 25863718 PMC4409557

[bib42] Fox M. D., & Raichle M. E. (2007). Spontaneous fluctuations in brain activity observed with functional magnetic resonance imaging. Nature Reviews Neuroscience, 8(9), 700–711. 17704812 10.1038/nrn2201

[bib43] Fukushima M., Betzel R. F., He Y., de Reus M. A., van den Heuvel M. P., Zuo X. N., & Sporns O. (2017). Fluctuations between high- and low-modularity topology in time-resolved functional connectivity. NeuroImage. 10.1016/j.neuroimage.2017.08.044 PMC6201264 28823827

[bib44] Geerligs L., Rubinov M., Cam C., & Henson R. N. (2015). State and Trait components of functional connectivity: Individual differences vary with mental state. Journal of Neuroscience, 35(41), 13949–13961. 10.1523/JNEUROSCI.1324-15.2015 26468196 PMC4604231

[bib45] Ghosh A., Rho Y., McIntosh A. R., Kotter R., & Jirsa V. K. (2008). Noise during rest enables the exploration of the brain’s dynamic repertoire. PLoS Computational Biology, 4(10), e1000196 10.1371/journal.pcbi.1000196 18846206 PMC2551736

[bib46] Giambra L. M. (1977). Daydreaming about the past. The time setting of spontaneous thought intrusions. Gerontologist, 17(1), 35–38. 844706 10.1093/geront/17.1.35

[bib47] Gilden D. L. (2001). Cognitive emissions of 1/f noise. Psychological Review, 108(1), 33–56. 11212631 10.1037/0033-295x.108.1.33

[bib48] Gonzalez-Castillo J., Hoy C. W., Handwerker D. A., Robinson M. E., Buchanan L. C., Saad Z. S., & Bandettini P. A. (2015). Tracking ongoing cognition in individuals using brief, whole-brain functional connectivity patterns. Proceedings of the National Academy of Sciences of the United States of America, 112(28), 8762–8767. 10.1073/pnas.1501242112 26124112 PMC4507216

[bib49] Gregory M. D., Robertson E. M., Manoach D. S., & Stickgold R. (2016). Thinking about a task is associated with increased connectivity in regions activated by task performance. Brain Connectivity, 6(2), 164–168. 10.1089/brain.2015.0386 26650337 PMC4779977

[bib50] Grooms J. K., Thompson G. J., Pan W. J., Billings J., Schumacher E. H., Epstein C. M., & Keilholz S. D. (2017). Infraslow electroencephalographic and dynamic resting state network activity. Brain Connectivity, 7(5), 265–280. 10.1089/brain.2017.0492 28462586 PMC5510044

[bib51] Gruber M. J., Ritchey M., Wang S. F., Doss M. K., & Ranganath C. (2016). Post-learning Hippocampal dynamics promote preferential retention of rewarding events. Neuron, 89(5), 1110–1120. 10.1016/j.neuron.2016.01.017 26875624 PMC4777629

[bib52] Hacker C. D., Snyder A. Z., Pahwa M., Corbetta M., & Leuthardt E. C. (2017). Frequency-specific electrophysiologic correlates of resting state fMRI networks. NeuroImage, 149, 446–457. 10.1016/j.neuroimage.2017.01.054 28159686 PMC5745814

[bib53] Haimovici A., Tagliazucchi E., Balenzuela P., & Laufs H. (2017). On wakefulness fluctuations as a source of BOLD functional connectivity dynamics. Scientific Reports, 7(1), 5908 10.1038/s41598-017-06389-4 28724928 PMC5517577

[bib54] Handwerker D. A., Roopchansingh V., Gonzalez-Castillo J., & Bandettini P. A. (2012). Periodic changes in fMRI connectivity. NeuroImage, 63(3), 1712–1719. 22796990 10.1016/j.neuroimage.2012.06.078 PMC4180175

[bib55] Harmelech T., & Malach R. (2013). Neurocognitive biases and the patterns of spontaneous correlations in the human cortex. Trends in Cognitive Sciences, 17(12), 606–615. 10.1016/j.tics.2013.09.014 24182697

[bib56] Harmelech T., Preminger S., Wertman E., & Malach R. (2013). The day-after effect: Long term, Hebbian-like restructuring of resting-state fMRI patterns induced by a single epoch of cortical activation. Journal of Neuroscience, 33(22), 9488–9497. 10.1523/JNEUROSCI.5911-12.2013 23719815 PMC6618560

[bib57] He B. J. (2013). Spontaneous and task-evoked brain activity negatively interact. Journal of Neuroscience, 33(11), 4672–4682. 10.1523/JNEUROSCI.2922-12.2013 23486941 PMC3637953

[bib58] He B. J., Snyder A. Z., Zempel J. M., Smyth M. D., & Raichle M. E. (2008). Electrophysiological correlates of the brains intrinsic large-scale functional architecture. Proceedings of the National Academy of Sciences of the United States of America, 105(41), 16039–16044. 10.1073/pnas.0807010105 18843113 PMC2564983

[bib59] Hermans E. J., Kanen J. W., Tambini A., Fernandez G., Davachi L., & Phelps E. A. (2017). Persistence of amygdala-hippocampal connectivity and multi-voxel correlation structures during awake rest after fear learning predicts long-term expression of fear. Cerebral Cortex, 27(5), 3028–3041. 10.1093/cercor/bhw145 27242028 PMC6059183

[bib60] Hesselmann G., Kell C. A., Eger E., & Kleinschmidt A. (2008). Spontaneous local variations in ongoing neural activity bias perceptual decisions. Proceedings of the National Academy of Sciences of the United States of America, 105(31), 10984–10989. 10.1073/pnas.0712043105 18664576 PMC2504783

[bib61] Hesselmann G., Kell C. A., & Kleinschmidt A. (2008). Ongoing activity fluctuations in hMT+ bias the perception of coherent visual motion. Journal of Neuroscience, 28(53), 14481–14485. 10.1523/JNEUROSCI.4398-08.2008 19118182 PMC6671252

[bib62] Hiltunen T., Kantola J., Abou Elseoud A., Lepola P., Suominen K., Starck T., … Palva J. M. (2014). Infra-slow EEG fluctuations are correlated with resting-state network dynamics in fMRI. Journal of Neuroscience, 34(2), 356–362. 10.1523/JNEUROSCI.0276-13.2014 24403137 PMC6608153

[bib63] Hindriks R., Adhikari M. H., Murayama Y., Ganzetti M., Mantini D., Logothetis N. K., & Deco G. (2016). Can sliding-window correlations reveal dynamic functional connectivity in resting-state fMRI? NeuroImage, 127, 242–256. 10.1016/j.neuroimage.2015.11.055 26631813 PMC4758830

[bib64] Honey C. J., Kotter R., Breakspear M., & Sporns O. (2007). Network structure of cerebral cortex shapes functional connectivity on multiple time scales. Proceedings of the National Academy of Sciences of the United States of America, 104(24), 10240–10245. 10.1073/pnas.0701519104 17548818 PMC1891224

[bib65] Hutchison R. M., & Morton J. B. (2015). Tracking the brains functional coupling dynamics over development. Journal of Neuroscience, 35(17), 6849–6859. 10.1523/JNEUROSCI.4638-14.2015 25926460 PMC6605187

[bib66] Hutchison R. M., Womelsdorf T., Allen E. A., Bandettini P. A., Calhoun V. D., Corbetta M., … Chang C. (2013). Dynamic functional connectivity: Promise, issues, and interpretations. NeuroImage, 80, 360–378. 10.1016/j.neuroimage.2013.05.079 23707587 PMC3807588

[bib67] Hyde J. S., & Li R. (2014). Functional connectivity in rat brain at 200 μm resolution. Brain Connectivity, 4(7), 470–480. 10.1089/brain.2014.0281 25112943 PMC4146383

[bib68] Ji D., & Wilson M. A. (2007). Coordinated memory replay in the visual cortex and hippocampus during sleep. Nature Neuroscience, 10(1), 100–107. 10.1038/nn1825 17173043

[bib69] Jones D. T., Vemuri P., Murphy M. C., Gunter J. L., Senjem M. L., Machulda M. M., … Jack C. R. Jr. (2012). Non-stationarity in the “resting brain’s” modular architecture. PLoS One, 7(6), e39731 10.1371/journal.pone.0039731 22761880 PMC3386248

[bib70] Kaiser R. H., Whitfield-Gabrieli S., Dillon D. G., Goer F., Beltzer M., Minkel J., … Pizzagalli D. A. (2016). Dynamic resting-state functional connectivity in major depression. Neuropsychopharmacology, 41(7), 1822–1830. 10.1038/npp.2015.352 26632990 PMC4869051

[bib71] Kane M. J., Brown L. H., McVay J. C., Silvia P. J., Myin-Germeys I., & Kwapil T. R. (2007). For whom the mind wanders, and when: an experience-sampling study of working memory and executive control in daily life. Psychological Science, 18(7), 614–621. 17614870 10.1111/j.1467-9280.2007.01948.x

[bib72] Kaplan R., Adhikari M. H., Hindriks R., Mantini D., Murayama Y., Logothetis N. K., & Deco G. (2016). Hippocampal sharp-wave ripples influence selective activation of the default mode network. Current Biology, 26(5), 686–691. 10.1016/j.cub.2016.01.017 26898464 PMC4791429

[bib73] Karapanagiotidis T., Bernhardt B. C., Jefferies E., & Smallwood J. (2017). Tracking thoughts: Exploring the neural architecture of mental time travel during mind-wandering. NeuroImage, 147, 272–281. 10.1016/j.neuroimage.2016.12.031 27989779

[bib74] Keilholz S. D. (2014). The neural basis of time-varying resting-state functional connectivity. Brain Connectivity, 4(10), 769–779. 10.1089/brain.2014.0250 24975024 PMC4268576

[bib75] Keilholz S. D., Magnuson M. E., Pan W. J., Willis M., & Thompson G. J. (2013). Dynamic properties of functional connectivity in the rodent. Brain Connectivity, 3(1), 31–40. 10.1089/brain.2012.0115 23106103 PMC3621313

[bib76] Keilholz S. D., Pan W. J., Billings J., Nezafati M., & Shakil S. (2017). Noise and non-neuronal contributions to the BOLD signal: Applications to and insights from animal studies. NeuroImage, 154, 267–281. 10.1016/j.neuroimage.2016.12.019 28017922 PMC5481494

[bib77] Keller C. J., Bickel S., Honey C. J., Groppe D. M., Entz L., Craddock R. C., … Mehta A. D. (2013). Neurophysiological investigation of spontaneous correlated and anticorrelated fluctuations of the bold signal. Journal of Neuroscience, 33(15), 6333–6342. 10.1523/JNEUROSCI.4837-12.2013 23575832 PMC3652257

[bib78] Killingsworth M. A., & Gilbert D. T. (2010). A wandering mind is an unhappy mind. Science, 330(6006), 932. 21071660 10.1126/science.1192439

[bib79] Kleinschmidt A., Sterzer P., & Rees G. (2012). Variability of perceptual multistability: From brain state to individual trait. Philosophical Transactions of the Royal Society of London, 367(1591), 988–1000. 10.1098/rstb.2011.0367 22371620 PMC3282312

[bib80] Klinger E., & Cox W. M. (1987). Dimensions of thought flow in everyday life. Imagination, Cognition and Personality, 7(2), 105–128.

[bib81] Konishi M., Brown K., Battaglini L., & Smallwood J. (2017). When attention wanders: Pupillometric signatures of fluctuations in external attention. Cognition, 168, 16–26. 10.1016/j.cognition.2017.06.006 28645038

[bib82] Krienen F. M., Yeo B. T., & Buckner R. L. (2014). Reconfigurable task-dependent functional coupling modes cluster around a core functional architecture. Philosophical Transactions of the Royal Society of London, 369(1653). 10.1098/rstb.2013.0526 PMC4150301 25180304

[bib83] Kucyi A. (2017). Just a thought: How mind-wandering is represented in dynamic brain connectivity. NeuroImage. 10.1016/j.neuroimage.2017.07.001 28684334

[bib84] Kucyi A., & Davis K. D. (2014). Dynamic functional connectivity of the default mode network tracks daydreaming. NeuroImage, 100C, 471–480. 10.1016/j.neuroimage.2014.06.044 24973603

[bib85] Kucyi A., Esterman M., Riley C. S., & Valera E. M. (2016). Spontaneous default network activity reflects behavioral variability independent of mind-wandering. Proceedings of the National Academy of Sciences of the United States of America, 113(48), 13899–13904. 10.1073/pnas.1611743113 27856733 PMC5137714

[bib86] Kucyi A., Hove M. J., Esterman M., Hutchison R. M., & Valera E. M. (2017). Dynamic brain network correlates of spontaneous fluctuations in attention. Cerebral Cortex, 27(3), 1831–1840. 10.1093/cercor/bhw029 26874182 PMC6317462

[bib87] Kucyi A., Salomons T. V., & Davis K. D. (2013). Mind wandering away from pain dynamically engages antinociceptive and default mode brain networks. Proceedings of the National Academy of Sciences of the United States of America, 110(46), 18692–18697. 24167282 10.1073/pnas.1312902110 PMC3832014

[bib88] Kudrimoti H. S., Barnes C. A., & McNaughton B. L. (1999). Reactivation of hippocampal cell assemblies: Effects of behavioral state, experience, and EEG dynamics. Journal of Neuroscience, 19(10), 4090–4101. 10234037 10.1523/JNEUROSCI.19-10-04090.1999 PMC6782694

[bib89] Lansink C. S., Goltstein P. M., Lankelma J. V., McNaughton B. L., & Pennartz C. M. (2009). Hippocampus leads ventral striatum in replay of place-reward information. PLoS Biology, 7(8), e1000173 10.1371/journal.pbio.1000173 19688032 PMC2717326

[bib90] Laumann T. O., Gordon E. M., Adeyemo B., Snyder A. Z., Joo S. J., Chen M. Y., … Petersen S. E. (2015). Functional system and areal organization of a highly sampled individual human brain. Neuron, 87(3), 657–670. 10.1016/j.neuron.2015.06.037 26212711 PMC4642864

[bib91] Laumann T. O., Snyder A. Z., Mitra A., Gordon E. M., Gratton C., Adeyemo B., … Petersen S. E. (2017). On the stability of BOLD fMRI correlations. Cerebral Cortex, 27(10), 4719–4732. 10.1093/cercor/bhw265 27591147 PMC6248456

[bib92] Leonardi N., & Van De Ville D. (2015). On spurious and real fluctuations of dynamic functional connectivity during rest. NeuroImage, 104, 430–436. 10.1016/j.neuroimage.2014.09.007 25234118

[bib93] Lewis C. M., Baldassarre A., Committeri G., Romani G. L., & Corbetta M. (2009). Learning sculpts the spontaneous activity of the resting human brain. Proceedings of the National Academy of Sciences of the United States of America, 106(41), 17558–17563. 10.1073/pnas.0902455106 19805061 PMC2762683

[bib94] Li X., Zhu D., Jiang X., Jin C., Zhang X., Guo L., … Liu T. (2014). Dynamic functional connectomics signatures for characterization and differentiation of PTSD patients. Human Brain Mapping, 35(4), 1761–1778. 10.1002/hbm.22290 23671011 PMC3928235

[bib95] Liegeois R., Laumann T. O., Snyder A. Z., Zhou J., & Yeo B. T. T. (2017). Interpreting temporal fluctuations in resting-state functional connectivity MRI. NeuroImage, 163, 437–455. 10.1016/j.neuroimage.2017.09.012 28916180

[bib96] Logothetis N. K., Eschenko O., Murayama Y., Augath M., Steudel T., Evrard H. C., … Oeltermann A. (2012). Hippocampal-cortical interaction during periods of subcortical silence. Nature, 491(7425), 547–553. 10.1038/nature11618 23172213

[bib97] Mackey A. P., Miller Singley A. T., & Bunge S. A. (2013). Intensive reasoning training alters patterns of brain connectivity at rest. Journal of Neuroscience, 33(11), 4796–4803. 10.1523/JNEUROSCI.4141-12.2013 23486950 PMC3657728

[bib98] Maillet D., & Schacter D. L. (2016). From mind wandering to involuntary retrieval: Age-related differences in spontaneous cognitive processes. Neuropsychologia, 80, 142–156. 10.1016/j.neuropsychologia.2015.11.017 26617263 PMC4698179

[bib99] Mittner M., Boekel W., Tucker A. M., Turner B. M., Heathcote A., & Forstmann B. U. (2014). When the brain takes a break: A model-based analysis of mind wandering. Journal of Neuroscience, 34(49), 16286–16295. 10.1523/JNEUROSCI.2062-14.2014 25471568 PMC4252543

[bib100] Monto S., Palva S., Voipio J., & Palva J. M. (2008). Very slow EEG fluctuations predict the dynamics of stimulus detection and oscillation amplitudes in humans. Journal of Neuroscience, 28(33), 8268–8272. 10.1523/JNEUROSCI.1910-08.2008 18701689 PMC6670577

[bib101] Murty V. P., Tompary A., Adcock R. A., & Davachi L. (2016). Selectivity in post-encoding connectivity with high-level visual cortex is associated with reward-motivated memory. Journal of Neuroscience. 10.1523/JNEUROSCI.4032-15.2016 PMC5242406 28100737

[bib102] Nadasdy Z., Hirase H., Czurko A., Csicsvari J., & Buzsaki G. (1999). Replay and time compression of recurring spike sequences in the hippocampus. Journal of Neuroscience, 19(21), 9497–9507. 10531452 10.1523/JNEUROSCI.19-21-09497.1999 PMC6782894

[bib103] Nir Y., Mukamel R., Dinstein I., Privman E., Harel M., Fisch L., … Malach R. (2008). Interhemispheric correlations of slow spontaneous neuronal fluctuations revealed in human sensory cortex. Nature Neuroscience, 11(9), 1100–1108. 19160509 10.1038/nn.2177 PMC2642673

[bib104] Palva J. M., & Palva S. (2011). Roles of multiscale brain activity fluctuations in shaping the variability and dynamics of psychophysical performance. Progress in Brain Research, 193, 335–350. 21854973 10.1016/B978-0-444-53839-0.00022-3

[bib105] Pan W. J., Thompson G., Magnuson M., Majeed W., Jaeger D., & Keilholz S. (2011). Broadband local field potentials correlate with spontaneous fluctuations in functional magnetic resonance imaging signals in the rat somatosensory cortex under isoflurane anesthesia. Brain Connectivity, 1(2), 119–131. 10.1089/brain.2011.0014 22433008 PMC3621847

[bib106] Pan W. J., Thompson G. J., Magnuson M. E., Jaeger D., & Keilholz S. (2013). Infraslow LFP correlates to resting-state fMRI BOLD signals. NeuroImage, 74, 288–297. 10.1016/j.neuroimage.2013.02.035 23481462 PMC3615090

[bib107] Pessoa L., & Padmala S. (2005). Quantitative prediction of perceptual decisions during near-threshold fear detection. Proceedings of the National Academy of Sciences of the United States of America, 102(15), 5612–5617. 10.1073/pnas.0500566102 15800041 PMC556244

[bib108] Ploner M., Lee M. C., Wiech K., Bingel U., & Tracey I. (2010). Prestimulus functional connectivity determines pain perception in humans. Proceedings of the National Academy of Sciences of the United States of America, 107(1), 355–360. 19948949 10.1073/pnas.0906186106 PMC2806712

[bib109] Poerio G. L., Sormaz M., Wang H. T., Margulies D., Jefferies E., & Smallwood J. (2017). The role of the default mode network in component processes underlying the wandering mind. Social Cognitive and Affective Neuroscience, 12(7), 1047–1062. 10.1093/scan/nsx041 28402561 PMC5490683

[bib110] Preti M. G., Bolton T. A., & Van De Ville D. (2016). The dynamic functional connectome: State-of-the-art and perspectives. NeuroImage. 10.1016/j.neuroimage.2016.12.061 28034766

[bib111] Raemaekers M., Schellekens W., van Wezel R. J., Petridou N., Kristo G., & Ramsey N. F. (2014). Patterns of resting state connectivity in human primary visual cortical areas: A 7T fMRI study. NeuroImage, 84, 911–921. 10.1016/j.neuroimage.2013.09.060 24099850

[bib112] Raichle M. E. (2015). The restless brain: How intrinsic activity organizes brain function. Philosophical Transactions of the Royal Society of London, 370(1668). 10.1098/rstb.2014.0172 PMC4387513 25823869

[bib113] Rashid B., Damaraju E., Pearlson G. D., & Calhoun V. D. (2014). Dynamic connectivity states estimated from resting fMRI identify differences among schizophrenia, bipolar disorder, and healthy control subjects. Frontiers in Human Neuroscience, 8, 897 10.3389/fnhum.2014.00897 25426048 PMC4224100

[bib114] Ratcliff R., Philiastides M. G., & Sajda P. (2009). Quality of evidence for perceptual decision making is indexed by trial-to-trial variability of the EEG. Proceedings of the National Academy of Sciences of the United States of America, 106(16), 6539–6544. 10.1073/pnas.0812589106 19342495 PMC2672543

[bib115] Ress D., Backus B. T., & Heeger D. J. (2000). Activity in primary visual cortex predicts performance in a visual detection task. Nature Neuroscience, 3(9), 940–945. 10.1038/78856 10966626

[bib116] Ritchey M., Wing E. A., LaBar K. S., & Cabeza R. (2013). Neural similarity between encoding and retrieval is related to memory via hippocampal interactions. Cerebral Cortex, 23(12), 2818–2828. 10.1093/cercor/bhs258 22967731 PMC3827709

[bib117] Rosenberg M. D., Finn E. S., Constable R. T., & Chun M. M. (2015). Predicting moment-to-moment attentional state. NeuroImage, 114, 249–256. 10.1016/j.neuroimage.2015.03.032 25800207

[bib118] Sadaghiani S., Hesselmann G., & Kleinschmidt A. (2009). Distributed and antagonistic contributions of ongoing activity fluctuations to auditory stimulus detection. Journal of Neuroscience, 29(42), 13410–13417. 19846728 10.1523/JNEUROSCI.2592-09.2009 PMC6665194

[bib119] Sadaghiani S., & Kleinschmidt A. (2013). Functional interactions between intrinsic brain activity and behavior. NeuroImage, 80, 379–386. 10.1016/j.neuroimage.2013.04.100 23643921

[bib120] Sadaghiani S., Poline J. B., Kleinschmidt A., & D’Esposito M. (2015). Ongoing dynamics in large-scale functional connectivity predict perception. Proceedings of the National Academy of Sciences of the United States of America, 112(27), 8463–8468. 10.1073/pnas.1420687112 26106164 PMC4500238

[bib121] Sami S., & Miall R. C. (2013). Graph network analysis of immediate motor-learning induced changes in resting state BOLD. Frontiers in Human Neuroscience, 7, 166 10.3389/fnhum.2013.00166 23720616 PMC3654214

[bib122] Sami S., Robertson E. M., & Miall R. C. (2014). The time course of task-specific memory consolidation effects in resting state networks. Journal of Neuroscience, 34(11), 3982–3992. 10.1523/JNEUROSCI.4341-13.2014 24623776 PMC3951697

[bib123] Schlichting M. L., & Preston A. R. (2014). Memory reactivation during rest supports upcoming learning of related content. Proceedings of the National Academy of Sciences of the United States of America, 111(44), 15845–15850. 10.1073/pnas.1404396111 25331890 PMC4226095

[bib124] Seli P., Risko E. F., & Smilek D. (2016). On the necessity of distinguishing between unintentional and intentional mind wandering. Psychological Science, 27(5), 685–691. 10.1177/0956797616634068 26993740

[bib125] Shakil S., Lee C. H., & Keilholz S. D. (2016). Evaluation of sliding window correlation performance for characterizing dynamic functional connectivity and brain states. NeuroImage, 133, 111–128. 10.1016/j.neuroimage.2016.02.074 26952197 PMC4889509

[bib126] Shen H., Li Z., Qin J., Liu Q., Lubin W., Zeng L. L., … Hu D. (2015). Changes in functional connectivity dynamics associated with vigilance network in taxi drivers. NeuroImage. 10.1016/j.neuroimage.2015.09.010 26363345

[bib127] Shine J. M., Bissett P. G., Bell P. T., Koyejo O., Balsters J. H., Gorgolewski K. J., … Poldrack R. A. (2016). The dynamics of functional brain networks: Integrated network states during cognitive task performance. Neuron, 92(2), 544–554. 10.1016/j.neuron.2016.09.018 27693256 PMC5073034

[bib128] Shine J. M., Koyejo O., & Poldrack R. A. (2016). Temporal metastates are associated with differential patterns of time-resolved connectivity, network topology, and attention. Proceedings of the National Academy of Sciences of the United States of America, 113(35), 9888–9891. 10.1073/pnas.1604898113 27528672 PMC5024627

[bib129] Shmuel A., & Leopold D. A. (2008). Neuronal correlates of spontaneous fluctuations in fMRI signals in monkey visual cortex: Implications for functional connectivity at rest. Human Brain Mapping, 29(7), 751–761. 10.1002/hbm.20580 18465799 PMC6870786

[bib130] Shulman R. G., Rothman D. L., Behar K. L., & Hyder F. (2004). Energetic basis of brain activity: Implications for neuroimaging. Trends in Neurosciences, 27(8), 489–495. 15271497 10.1016/j.tins.2004.06.005

[bib131] Siegel J. S., Mitra A., Laumann T. O., Seitzman B. A., Raichle M., Corbetta M., & Snyder A. Z. (2017). Data quality influences observed links between functional connectivity and behavior. Cerebral Cortex, 27(9), 4492–4502. 10.1093/cercor/bhw253 27550863 PMC6410500

[bib132] Simony E., Honey C. J., Chen J., Lositsky O., Yeshurun Y., Wiesel A., & Hasson U. (2016). Dynamic reconfiguration of the default mode network during narrative comprehension. Nature Communications, 7, 12141 10.1038/ncomms12141 PMC4960303 27424918

[bib133] Smallwood J. (2011). The footprints of a wandering mind: Further examination of the time course of an attentional lapse. Cognitive Neuroscience, 2(2), 91–97. 10.1080/17588928.2010.537746 24168478

[bib134] Smallwood J. (2013). Distinguishing how from why the mind wanders: A process-occurrence framework for self-generated mental activity. Psychological Bulletin, 139(3), 519–535. 10.1037/a0030010 23607430

[bib135] Smallwood J., Brown K. S., Tipper C., Giesbrecht B., Franklin M. S., Mrazek M. D., … Schooler J. W. (2011). Pupillometric evidence for the decoupling of attention from perceptual input during offline thought. PLoS One, 6(3), e18298 10.1371/journal.pone.0018298 21464969 PMC3064669

[bib136] Smallwood J., Davies J. B., Heim D., Finnigan F., Sudberry M., O’Connor R., & Obonsawin M. (2004). Subjective experience and the attentional lapse: Task engagement and disengagement during sustained attention. Consciousness and Cognition, 13(4), 657–690. 10.1016/j.concog.2004.06.003 15522626

[bib137] Smallwood J., Karapanagiotidis T., Ruby F., Medea B., de Caso I., Konishi M., … Jefferies E. (2016). Representing representation: Integration between the Temporal lobe and the posterior cingulate influences the content and form of spontaneous thought. PLoS One, 11(4), e0152272 10.1371/journal.pone.0152272 27045292 PMC4821638

[bib138] Smallwood J., McSpadden M., Luus B., & Schooler J. (2008). Segmenting the stream of consciousness: The psychological correlates of temporal structures in the time series data of a continuous performance task. Brain and Cognition, 66(1), 50–56. 10.1016/j.bandc.2007.05.004 17614178

[bib139] Smallwood J., & Schooler J. W. (2006). The restless mind. Psychological Bulletin, 132(6), 946–958. 10.1037/0033-2909.132.6.946 17073528

[bib140] Smallwood J., & Schooler J. W. (2015). The science of mind wandering: Empirically navigating the stream of consciousness. Annual Review of Psychology, 66, 487–518. 10.1146/annurev-psych-010814-015331 25293689

[bib141] Sonuga-Barke E. J., & Castellanos F. X. (2007). Spontaneous attentional fluctuations in impaired states and pathological conditions: A neurobiological hypothesis. Neuroscience & Biobehavioral Reviews, 31(7), 977–986. 10.1016/j.neubiorev.2007.02.005 17445893

[bib142] Sporns O. (2010). Networks of the Brain. Cambridge, MA: MIT Press.

[bib143] Staresina B. P., Alink A., Kriegeskorte N., & Henson R. N. (2013). Awake reactivation predicts memory in humans. Proceedings of the National Academy of Sciences of the United States of America, 110(52), 21159–21164. 10.1073/pnas.1311989110 24324174 PMC3876238

[bib144] Stawarczyk D., Majerus S., Maj M., Van der Linden M., & D’Argembeau A. (2011). Mind-wandering: Phenomenology and function as assessed with a novel experience sampling method. Acta Psychologica (Amst), 136(3), 370–381. 10.1016/j.actpsy.2011.01.002 21349473

[bib145] Tagliazucchi E., & van Someren E. J. W. (2017). The large-scale functional connectivity correlates of consciousness and arousal during the healthy and pathological human sleep cycle. NeuroImage. 10.1016/j.neuroimage.2017.06.026 28619656

[bib146] Tagliazucchi E., von Wegner F., Morzelewski A., Brodbeck V., & Laufs H. (2012). Dynamic BOLD functional connectivity in humans and its electrophysiological correlates. Frontiers in Human Neuroscience, 6, 339 10.3389/fnhum.2012.00339 23293596 PMC3531919

[bib147] Tambini A., & Davachi L. (2013). Persistence of hippocampal multivoxel patterns into postencoding rest is related to memory. Proceedings of the National Academy of Sciences of the United States of America, 110(48), 19591–19596. 10.1073/pnas.1308499110 24218550 PMC3845130

[bib148] Tambini A., Ketz N., & Davachi L. (2010). Enhanced brain correlations during rest are related to memory for recent experiences. Neuron, 65(2), 280–290. 10.1016/j.neuron.2010.01.001 20152133 PMC3287976

[bib149] Thompson G. J., Magnuson M. E., Merritt M. D., Schwarb H., Pan W. J., McKinley A., … Keilholz S. D. (2013). Short-time windows of correlation between large-scale functional brain networks predict vigilance intraindividually and interindividually. Human Brain Mapping, 34(12), 3280–3298. 10.1002/hbm.22140 22736565 PMC6870033

[bib150] Thompson G. J., Merritt M. D., Pan W. J., Magnuson M. E., Grooms J. K., Jaeger D., & Keilholz S. D. (2013). Neural correlates of time-varying functional connectivity in the rat. NeuroImage, 83C, 826–836. 10.1016/j.neuroimage.2013.07.036 PMC3815981 23876248

[bib151] Thompson G. J., Pan W. J., Billings J. C., Grooms J. K., Shakil S., Jaeger D., & Keilholz S. D. (2014). Phase-amplitude coupling and infraslow (<1 Hz) frequencies in the rat brain: Relationship to resting state fMRI. Frontiers in Integrative Neuroscience, 8, 41 10.3389/fnint.2014.00041 24904325 PMC4034045

[bib152] Thompson G. J., Pan W. J., & Keilholz S. D. (2015). Different dynamic resting state fMRI patterns are linked to different frequencies of neural activity. Journal of Neurophysiology, 114(1), 114–124. 10.1152/jn.00235.2015 26041826 PMC4507971

[bib153] Thompson G. J., Pan W. J., Magnuson M. E., Jaeger D., & Keilholz S. D. (2014). Quasi-periodic patterns (QPP): Large-scale dynamics in resting state fMRI that correlate with local infraslow electrical activity. NeuroImage, 84, 1018–1031. 10.1016/j.neuroimage.2013.09.029 24071524 PMC3869452

[bib154] Thompson G. J., Riedl V., Grimmer T., Drzezga A., Herman P., & Hyder F. (2016). The whole-brain “global” signal from resting state fMRI as a potential biomarker of quantitative state changes in glucose metabolism. Brain Connectivity, 6(6), 435–447. 10.1089/brain.2015.0394 27029438 PMC4976226

[bib155] Tompary A., Duncan K., & Davachi L. (2015). Consolidation of associative and item memory is related to post-encoding functional connectivity between the ventral tegmental area and different medial temporal lobe subregions during an unrelated task. Journal of Neuroscience, 35(19), 7326–7331. 10.1523/JNEUROSCI.4816-14.2015 25972163 PMC4429149

[bib156] Tusche A., Smallwood J., Bernhardt B. C., & Singer T. (2014). Classifying the wandering mind: Revealing the affective content of thoughts during task-free rest periods. NeuroImage, 97, 107–116. 10.1016/j.neuroimage.2014.03.076 24705200

[bib157] Vanhaudenhuyse A., Demertzi A., Schabus M., Noirhomme Q., Bredart S., Boly M., … Laureys S. (2011). Two distinct neuronal networks mediate the awareness of environment and of self. Journal of Cognitive Neuroscience, 23(3), 570–578. 20515407 10.1162/jocn.2010.21488

[bib158] Verplanck W. S., Collier G. H., & Cotton J. W. (1952). Nonindependence of successive responses in measurements of the visual threshold. Journal of Experimental Psychology, 44(4), 273–282. 13000069 10.1037/h0054948

[bib159] Vincent J. L., Patel G. H., Fox M. D., Snyder A. Z., Baker J. T., Van Essen D. C., … Raichle M. E. (2007). Intrinsic functional architecture in the anaesthetized monkey brain. Nature, 447(7140), 83–86. 10.1038/nature05758 17476267

[bib160] Wang C., Ong J. L., Patanaik A., Zhou J., & Chee M. W. (2016). Spontaneous eyelid closures link vigilance fluctuation with fMRI dynamic connectivity states. Proceedings of the National Academy of Sciences of the United States of America, 113(34), 9653–9658. 10.1073/pnas.1523980113 27512040 PMC5003283

[bib161] Wang D., Buckner R. L., Fox M. D., Holt D. J., Holmes A. J., Stoecklein S., … Liu H. (2015). Parcellating cortical functional networks in individuals. Nature Neuroscience, 18(12), 1853–1860. 10.1038/nn.4164 26551545 PMC4661084

[bib162] Weisz N., Wuhle A., Monittola G., Demarchi G., Frey J., Popov T., & Braun C. (2014). Prestimulus oscillatory power and connectivity patterns predispose conscious somatosensory perception. Proceedings of the National Academy of Sciences of the United States of America, 111(4), E417–425. 10.1073/pnas.1317267111 24474792 PMC3910583

[bib163] Wong C. W., DeYoung P. N., & Liu T. T. (2016). Differences in the resting-state fMRI global signal amplitude between the eyes open and eyes closed states are related to changes in EEG vigilance. NeuroImage, 124(Pt A), 24–31. 10.1016/j.neuroimage.2015.08.053 26327245

[bib164] Yang Z., Craddock R. C., Margulies D. S., Yan C. G., & Milham M. P. (2014). Common intrinsic connectivity states among posteromedial cortex subdivisions: Insights from analysis of temporal dynamics. NeuroImage, 93 Pt. 1, 124–137. 10.1016/j.neuroimage.2014.02.014 24560717 PMC4010223

